# Preliminary development of recommendations for the inclusion of patient-reported outcome measures in clinical quality registries

**DOI:** 10.1186/s12913-022-07657-4

**Published:** 2022-03-01

**Authors:** Rasa Ruseckaite, Ashika D. Maharaj, Joanne Dean, Karolina Krysinska, Ilana N. Ackerman, Angela L. Brennan, Ljoudmila Busija, Helen Carter, Arul Earnest, Christopher B. Forrest, Ian A. Harris, Janet Sansoni, Susannah Ahern

**Affiliations:** 1 Department of Epidemiology and Preventive Medicine, Monash University, Melbourne, Victoria Australia; 2 Centre of Cardiovascular Research and Education in Therapeutics, Monash University, Melbourne, Victoria 3004 Australia; 3 Australian Stroke Clinical Registry, The Florey Institute of Neuroscience & Mental Health, Melbourne, Victoria Australia; 4 Children’s Hospital of Philadelphia, Philadelphia, PA USA; 5 Whitlam Orthopaedic Research Centre, Ingham Institute for Applied Medical Research, Sydney, Australia; 6 South Western Sydney Clinical School, University of New South Wales, Sydney, New South Wales Australia; 7 Centre for Health Service Development, Australian Health Services Research Institute, University of Wollongong, Wollongong, New South Wales Australia

**Keywords:** PROMs, Quality of life, Registry, Outcomes, Recommendations, Delphi

## Abstract

**Background:**

Clinical quality registries (CQRs) monitor compliance against optimal practice and provide feedback to the clinical community and wider stakeholder groups. Despite a number of CQRs having incorporated the patient perspective to support the evaluation of healthcare delivery, no recommendations for inclusion of patient-reported outcome measures (PROMs) in CQRs exist. The aim of this study was to develop a core set of recommendations for PROMs inclusion of in CQRs.

**Method:**

An online two-round Delphi survey was performed among CQR data custodians, quality of life researchers, biostatisticians and clinicians largely recruited in Australia. A list of statements for the recommendations was identified from a literature and survey of the Australian registries conducted in 2019. The statements were grouped into the following domains: rationale, setting, ethics, instrument, administration, data management, statistical methods, and feedback and reporting. Eighteen experts were invited to participate, 11 agreed to undertake the first online survey (round 1). Of these, nine experts completed the online survey for round 2.

**Results:**

From 117 statements presented to the Delphi panel in round 1, a total of 72 recommendations (55 from round 1 and 17 from round 2) with median importance (MI) ≥ 7 and disagreement index (DI) < 1 were proposed for inclusion into the final draft set and were reviewed by the project team. Recommendations were refined for clarity and to read as stand-alone statements. Ten overlapped conceptually and, therefore, were merged to reduce repetition. The final 62 recommendations were sent for review to the panel members for their feedback, which was incorporated into the final set.

**Conclusion:**

This is the first study to develop preliminary recommendations for PROMs inclusion in CQRs. Recommendations for PROMs implementation are critically important for registries to assure meaningful PROMs data capture, use, interpretation, and reporting to improve health outcomes and healthcare value.

## Background

Patient reported outcome measures (PROMs) are designed to assess various dimensions of a person’s health and well-being from the perspective of the individual themselves. Beyond assessing treatment effectiveness in the context of clinical trials and other research activities, PROMs have been used in clinical practice, supporting patient-centred care and shared clinical decision making [1]. The use of PROMs has been demonstrated to: 1) enhance quality of care and decision making in routine care for cardiovascular disease [2], and 2) identify clinical best practice and improve average health outcomes through tracking health and disseminating outcomes from clinical quality registries (CQRs) [3].

CQRs are organisations that systematically monitor the quality of healthcare within specific clinical domains by routinely collecting, analysing and reporting health-related information [4]. They use predefined indicators to assess variation across structural, process and outcome measures in order to benchmark quality of care [5]. CQRs have received increasing attention as a means of improving quality and reducing the cost of health and medical care, through identifying variations in clinical practice and care, and assessing the uptake of effective treatment [6, 7].

Data collected using PROMs and integrated in a feedback mechanism within a CQR can be used to track the benefit of clinical interventions with the potential to improve shared decision-making and treatment outcomes for patients [8]. The inclusion of PROMs in CQRs offers numerous advantages [9]. First, incorporation of the patient voice regarding their lived experiences ensures that health outcome measurements of care are patient-centred. Further, symptom burden and quality of life (QoL) are dynamic variables that cannot be recreated accurately through retrospection; they are essentially lost if not captured “in the moment”. For this reason, routine, systematic, and longitudinal collection of PROMs have been recommended as a standard aspect of clinical practice [10, 11]. Likewise, longitudinal collection of PROMs, in addition to clinician derived medical data in CQRs, can improve understanding of the trajectory of an individual patient’s symptom burden and QoL over the course of the disease or treatment. This can inform clinicians of the variability between patient groups, provide information on the value patients place on their health status and to predict patient outcomes [9].

Numerous guides and recommendations were developed to promote patient-centered care and PROMs use in clinical practice. Users’ guide to integrating patient-reported outcomes in electronic health records provides recommendations for integrating PROMs into electronic health records, thus enabling use of outcome data for multiple applications [12]. The International Society for Quality of Life Research (ISOQOL) guide [13] provides options for how to select PROM measures, as well as guidance on data collection and reporting in clinical practice. The purpose of this guide is to help clinicians who are interested in using PROMs in their clinical practice as a tool in patient management. Similarly, guidelines have been developed for inclusion of PROMs in clinical trial protocols [14].

The above listed guides provide recommendations for clinicians capturing PROMs data in clinical practice and to tailor care to individual needs. Clinical registries play an increasingly important role as a stimulus for quality improvement by providing high-quality data and analyses that are respected by clinicians [15, 16]. PROMs in CQRs are used for reporting and benchmarking purposes [16, 17]. Implementation of lessons learned from CQRs that include PROMs will assure patients achieve optimal management of the disease and functional gain with minimal adverse events [9]. Although some registries have included PROMs as part of their current practice [18, 19], widespread adoption of PROMs as a key component in CQRs is yet to occur. PROMs are increasingly being introduced into CQRs in Australia. For example, the Victorian Orthopaedic Trauma Outcomes Registry [20] and the Prostate Cancer Outcomes Registry – Victoria [21] both collect PROMs at a time of clinical stability. PROMs data collection is currently being considered by the Australian and New Zealand Thyroid Cancer Registry (ANZTCR) [22] and the Australasian Pelvic Floor Procedure Registry (APFPR) [23].

There are a range of methodological considerations required for PROMs implementation in registries to ensure they provide the most benefit and deliver measurable and actionable outcome data, particularly as incorporating PROMs into CQRs is likely to be costly and time-consuming. Clear recommendations are needed to support ethical, effective, and transparent use of PROMs collected across all CQRs [9, 24].

The aim of this project was to develop, using a Delphi method, a set of recommendations for PROMs inclusion in a CQR setting. This publication is the second in a series describing the development of evidence-informed guidelines for PROMs inclusion within CQRs in Australia. The preceding study developed a conceptual framework for the inclusion of PROMs in CQRs, which classified findings, from both the literature and the survey of 66 Australian registries, into broad categories ranging from initial development to outcome dissemination providing the structure for development of recommendations, engaging national and international leaders in health-related QoL research, clinicians, researchers, patient advocates and consumers [25].

## Methods

### Study design

An online classical Delphi method consisting of two survey rounds was employed in this study. Surveys were distributed using the secure Qualtrics survey software (https://www.qualtrics.com).

The Delphi approach was chosen as it can be delivered remotely in a short time frame without the need to convene meetings. It also enables researchers to collect the opinions of a range of different individuals with differing areas of expertise which was desirable in this setting survey [26, 27].

### Development of recommendations for Delphi panel

A list of preliminary statements for the recommendations was based on the literature review and a survey of existing Australian registries, conducted in 2019 [16]. A total of 3661 articles published between July 2018 and September 2018 were identified. Following title and abstract screening of studies that focussed on lessons learnt, advantages and disadvantages, guidelines and recommendations for PROMs inclusion in CQRs, 10 full text articles were assessed.

An initial survey of the registries aimed to gain a baseline understanding of the purpose of collecting PROMs, the principles driving their collection, patient coverage, and the manner of application by Australian registries who were identified as early adopters. Of the 66 Australian registries identified in the survey, only nineteen (29%) confirmed that they collected PROMs.

The statements arising from the literature review and survey responses were grouped into a conceptual framework that included the following domains: rationale, setting, ethics, instrument, administration, data management, statistical methods, and feedback/reporting of the PROMs data [16]. Each of the domains were further divided into categories, with the relevant recommendations. The list of potential recommendations was revised for clarity by the project team, reworded for standardisation and consistency and presented to the Delphi panel.

### Selection of panel members

A Delphi study was performed among CQR data custodians, QoL and PROMs researchers, biostatisticians and clinicians. Purposive sampling was used to identify Delphi panel members who all collectively had excellent contemporary understanding of PROMs, QoL measures and CQRs. Australian participants from a broad range of disciplines were identified through various professional networks and societies. Experts from the ISOQOL were also invited to participate. All potential participants to the Delphi panel received an electronic invitation to be involved in the study. Commitment to contribute to at least two rounds was requested when agreeing to participate in this process. Non-responders received up to two reminders prior to the date of closure.

Invitation to the first Delphi round was sent on the 19th July, 2019, and the second round was conducted on the 8th November, 2019.

### Data analysis

#### Panel ratings

The panel was asked to use a Likert scale ranging from 1 (not important) to 9 (very important) to rank the importance of a proposed statement. There was the option of ‘unable to comment’ if participants felt that they had inadequate knowledge or experience to rate a proposed statement. Members of the Delphi panel were also able to provide their feedback on each of the statements and propose new recommendations.

The results were analysed using Excel 2013 to calculate the median importance (MI) ranging from 1 to 9 and disagreement index (DI). The DI is a continuous scale that measures the variation in expert ratings. Based on the RAND method [26] DI of 0 represents complete agreement whereas DI ≥ 1 indicates significant disagreement or lack of consensus. If the DI exceeds 1, then the distribution meets criteria for extreme variation in ratings. The DI is calculated by using a standard published equation [26]. An ‘unable to comment’ response was excluded from the calculations. Statements with a MI of ≥7 and a DI < 1 progressed to a set of candidate statements. The Delphi panel was able to refine the wording of statements and to propose new ones, supported by evidence, that were felt to be important for implementing PROMs in CQRs. The results were sent to the Delphi panel in the second round. The process that was followed in the second round was the same as in the first round.

#### Post-hoc analysis

The results from the second round were then reviewed by the project team. Ranking, scores of importance, expert feedback on wording and their other comments were considered. Statements that were rated as DI ≥ 1 and MI ≤ 7 in both rounds were removed. In addition, statements with similar meanings were consolidated into a single statement. A final draft of statements was generated from both Delphi rounds and distributed to the members of the Delphi panel for the final review.

## Results

### Delphi rounds

Of the 18 (12 from Australia and six international) experts invited to participate in this study, 11 (eight female) agreed to undertake the first online survey (round one). Ten experts were from Australia, and one QoL expert and clinician was from the United States of America. Of these, nine experts completed the online survey for the second round.

In the first round, members of the Delphi panel were presented with a list of 117 statements for recommendations, accompanied by a supplementary document that included information about the process. Of the 117 potential statements presented to the panel in the first round, 55 (47%) statements were rated as very important (MI ≥ 7) with low disagreement (DI ≤ 1). These statements were automatically included into the final set. Eleven (9%) statements were rated as unimportant and were excluded from the further evaluation. The remaining 51 (44%) statements did not reach agreement (DI ≥ 1) (Table 1). At the conclusion of the first round, ten new statements were suggested for the second round, and seven existing statements that contained an additional idea or concept worthy of their own were recommended to be separated. In total, 68 items were presented to the panel in the second round.

**Table 1:**
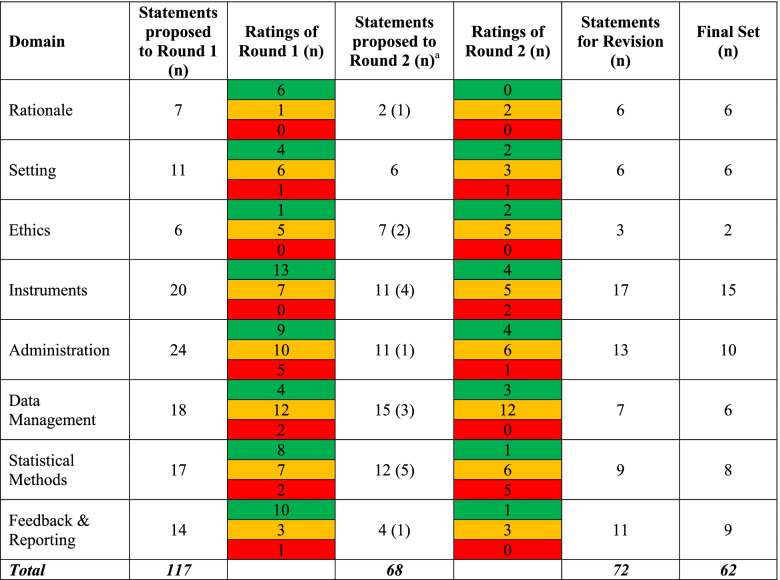
Delphi panel summary results

^a^number in parenthesis denotes new statements proposed for Round 2. Green colour denotes statements rated as very important (MI ≥ 7 & DI < 1), amber - statements with disagreement (DI ≥ 1), red - statements rated as not important MI ≤ 5).

At the conclusion of the second round, 17 (25%) statements were deemed very important (MI ≥ 7) with low disagreement (DI < 1), 42 (62%) statements did not reach agreement, and the remaining nine (13%) were rated as non-important. Statements that did not reach importance in both rounds (were rated as DI ≥ 1 and MI ≤ 7) were removed.

A total of 72 statements (55 from the first round and 17 from the second round) with MI ≥ 7 and DI < 1 were proposed for inclusion into the final set and were reviewed by the project team. Statements were refined, reworded and further abbreviated for clarity and to read as stand-alone. Ten recommendations (one each for the “Ethics”, “Data Management” and “Statistical Management” domains, two each for the “Instruments” and “Feedback & Reporting” domains, and three from for the “Administration” domain) overlapped conceptually and, therefore, were merged to reduce repetition. This resulted in a reduction in the number of recommendations within each domain. The final set of 62 recommendations were sent for review to the Delphi panel members for their feedback, which was collated in the final set (Table 2).

**Table 2:** Final set of recommendations for PROMs inclusions in CQRs

	**RATIONALE**	**MI**	**DI**
**1.1**	**Purpose of collecting PROMs**		
1.1.1	In registries PROMs may be collected for a variety of purposes, such as: - to **promote patient engagement in their treatment** including measuring relevant symptoms/adverse events/treatment outcomes and monitoring changes over time; - to **inform** patient's choice of treatment or **access to quality of care**; - to measure quality of care and **patient outcomes** in the real world; - to **facilitate shared decision making** between clinician and patient, and to support patient centred care; - to **inform models of care**; - to **support health service improvements** by identifying variation in care; - to **identify subgroups of patients** with persistent adverse outcomes indicative of increased risk of procedure/treatment/device failure; - to identify **patients with the greatest need** to support the allocation of healthcare resources; - to **measure burden of disease**; - to support **post-marketing surveillance activities**; - to guide the specialist community to **determine best practice**.	8	0.54
1.1.2	PROMs may be useful as a measure of **positive** outcomes such as pain relief and improved function and as a marker of risk for **negative** outcomes such as persistent pain or reduced function.	8	0.49
1.1.3	Use of PROMs should be driven by **outcomes** capable of contributing to improving patient care **that can be met through patient reporting**. Consensus of key objectives should occur before the design stage.	8	0.49
**1.2**	**Stakeholders**		
1.2.1	**Stakeholders** with an interest in PROMs may include patients/consumers, clinicians, funders, insurers, health services, Departments of Health/Government, policy makers, academics and/or others with relevant expertise/experience.	7	0.75
1.2.2	Stakeholders should assist in the **development** of a PROMs framework encompassing the aim, purpose, scope, and infrastructure, selection of the PROM and data collection strategies.	7	0.37
1.2.3	Different stakeholders may identify **various primary goals** for use of the PROMs (*Please see 1.1.1 for further details*).	7	0.74
	**SETTING**		
**2.1**	**Implementation**		
2.1.1	Registries should develop a **holistic framework** to define and guide PROMs implementation (e.g. who, when, where, what and how), to identify and address potential barriers for implementation (e.g. patient recruitment, patient language, operational, cultural, resourcing, cost, expertise, appropriate instruments) and to allocate appropriate resources and expertise.	7	0.42
2.1.2	Consideration should be given to **piloting** PROMs implementation before the full rollout to assess feasibility from the patient, clinician and/or system perspective.	9	0.49
**2.2**	**Population and sample size**		
2.2.1	It may be a cost-effective strategy to **target populations** within the registry that are most in need and establishing where PROMs can be most usefully applied, e.g. recurrence following removal of a high-risk cancer.	7	1.50
2.2.2	When selecting a baseline PROM, consideration should be given to a screening process to identify the **eligible population** prior to the intervention.	7	0.72
2.2.3	Administration of **baseline PROMs** should include an assessment prior to an intervention where possible, and to provide a reference to PROMs assessments post intervention.	8	0.84
2.2.4	Depending on the purpose of data collection, PROMs may be collected from the **whole** population or a particular **sample** population.	8	0.33
	**ETHICS**		
**3.1**	**Patient consent**		
3.1.1	Information about the PROMs should be provided to participants using a method **approved by an ethics committee**. This information should include the benefits and risks of participation, and the process of withdrawal from participation.	8	0.75
3.1.2	Depending on the jurisdiction and institutional ethics review, **PROMs may require an opt-in or opt-out approach.**	8	0.33
	**INSTRUMENTS**		
**4.1**	**Consumer engagement**		
4.1.1	**Patients should be involved** in setting PROMs objectives and instrument choice, including the development and validation of new instruments, as well as use of PROMs data.	7	0.75
4.1.2	Response rates may be optimised by **providing patients with information on how data will be used** and the goals/aims for PROMs.	7	0.56
4.1.3	PROMs data collection should consider instrument type and mode of administration for **patients with special needs or disabilities.**	7	0.54
4.1.4	PROMs data collection should consider **patient preferences** for providing PROMs responses, including multiple modes of administration, if possible.	7	0.87
**4.2**	**New/Existing**		
4.2.1	PROMs selection should commence with a **literature review** to identify the range and frequency of use of existing validated instruments associated with the device/procedure/diagnosis/treatment. PROMs selection should meet the purpose of implementation and reflect the identified outcome of interest.	8	0.75
4.2.2	Any PROM selected may be an **existing** and validated tool, an **abbreviated** or amended version of an existing tool, or a **new** tool developed within the registry space, if existing tools are not available or adaptable.	7	0.74
4.2.3	New and abbreviated PROMs need to be **evaluated for validity** before being used to measure and report on outcomes.	8	0.51
4.2.4	PROMs with a clear **scoring system** should be used to maximise ease of use, administration and capacity to capture poor outcomes, if needed.	7	0.65
4.2.5	PROMs may be translated into **multiple languages** depending on the availability of validated translated versions and the cost of implementation.	7	0.45
4.2.6	A PROM **response scale** should consider a variety of response options (e.g. very dissatisfied, dissatisfied, neutral, satisfied, and very satisfied) to enable clear interpretation.	7	0.53
4.2.7	**PROMs selection** should be based on recommendations by experts in the field *including patients with lived experience of the disease/condition*, instrument reliability and validity, global standards (e.g. ICHOM or ISOQOL), completion time, costs and patient burden.	7	0.75
**4.3**	**Generic/Specific**		
4.3.1	Registries should consider using **item banks** (e.g. PROMIS, PROQOLID, EUROQOL, etc.), to access items that are individually validated and can be mixed and matched according to the overall purpose of PROM implementation.	7	0.51
4.3.2	Registries should consider using both **general and specific PROMs.** Generic PROMs such as SF-12 and EQ-5D may be useful in a registry setting for global health, research and policy purposes to compare against outcomes from other populations or healthcare interventions, and over time. Disease-/condition-/procedure-specific PROMs have greater clinical utility related to particular conditions, treatments and procedures.	7	0.89
4.3.3	**More than one instrument** may be required to meet the objective(s) of the PROMs data collection.	8	0.75
4.3.4	The number of instruments used and the number of items included in a specific PROMs data collection tool should be **minimised** without losing the essential constructs/domains related to the output of interest (especially for registries with large populations in order to maximise a response rate).	8	0.49
	**ADMINISTRATION**		
**5.1**	**Timing and Frequency**		
5.1.1	Based on discipline-specific clinical best practice and evidence, PROMs should be administered at various **time points** (e.g. baseline, single or multiple).	8	0.81
5.1.2	The length of **PROM data collection tools** and numbers of data collection points should consider patient and administrative burden.	8	0.49
5.1.3	PROMs data collection should consider patient’s burden in relation to the number and timing of **reminders** and the mode of administration.	7	0.75
5.1.4	Processes should be developed to avoid sending follow-up PROMs to **deceased** patients or patients who have **withdrawn their informed consent**.	9	0.29
**5.2**	**Modes and Methods**		
5.2.1	PROMs **data collection plan** should outline the mode(s) of administration (e.g. paper, telephone, electronic, other) and setting (e.g., clinic, home, other).	7	0.75
5.2.2	PROMs may be administered via **multiple methods** to increase response rate.	7	0.94
5.2.3	**Evaluation** of the PROMs program should be undertaken periodically and include feedback from patients, clinicians and other stakeholders.	8	0.42
5.2.4	Mode of PROMs administration should take into consideration **patient factors,** such as the age, gender and digital literacy.	7	0.75
5.2.5	**Computer adaptive testing systems** should be considered to minimise patient’s time and data entry burden.	7	0.75
5.2.6	PROMs **collection at the moment of contact with a clinician/service** encourages patient participation and maximises capture of complete data.	7	0.78
	**DATA MANAGEMENT**		
**6.1**	**Entry and Quality checks**		
6.1.1	PROMs data management should consider issues of data security, **information governance**, and availability of technology for data collection.	9	0.59
6.1.2	**Data management protocols** should include plans for data entry, coding, security, and storage, including any related processes to promote data quality (e.g. double data entry, range checks for data values, etc.)	8	0.54
6.1.3	Registries should provide **data management protocols** and training to assist staff in PROMs administration, data collection and data entry.	8	0.41
**6.2**	**IT design/Storage**		
6.2.1	**PROMs IT modules** should be designed to support PROM completion e.g. send regular email or phone reminders, validation checks for missing data prior to submission, store the completed data with the date of completion.	7	0.64
6.2.2	PROMs IT modules should be able to **calculate PROM scores** and to extract data from the database.	7	0.81
6.2.3	Appropriate strategy should be developed for **managing missing PROMs data** (e.g. missing items vs. non-response).	7	0.51
	**STATISTICAL METHODS**		
**7.1**	**PROMs Analysis**		
7.1.1	**Biostatisticians and/or epidemiologists** should be involved in processing and reporting PROMs data.	8	0.33
7.1.2	**Statistical methods** used for PROMs data analysis should be clearly described.	8	0.33
7.1.3	The volume, nature, and management of missing PROMs data should be described (e.g. **approach to imputation and sensitivity analyses).**	8	0.61
7.1.4	Clinical or sociodemographic differences between **respondent and non-respondent populations** should be reported to help explain possible variation in PROMs findings.	8	0.50
7.1.5	When a PROM is a primary outcome of interest, both **baseline and follow-up** information should be reported.	8	0.88
7.1.6	**Risk adjustment** should be undertaken to control for the role of confounding and case mix in PROMs data analysis, and adjustment for confounding and case-mix factors should be guided by causal knowledge.	7	0.67
7.1.7	**Confounding and case-mix factors** may be adjusted (1) in the design (e.g., restriction or matching techniques such as propensity or radius) or (2) in the analysis (e.g., inverse probability weighting, stratification, restriction).	7	0.79
7.1.8	**Real-time analyses** can provide PROMS data for shared decision-making in clinical practice.	7	0.65
	**FEEDBACK AND REPORTING**		
**8.1**	**Dissemination**		
8.1.1	The reporting of PROMs results should highlight the benefit of improving care for existing and/or future patients. Output should be published in a range of formats to **reach a broad range of stakeholders**.	8	0.48
8.1.2	PROMs outputs should be reported as aggregated data that maintains **confidentiality of participants**. It may be used to compare patient group results against the entire cohort, or used for comparative purposes including benchmarking outcomes, such as variation between two sites or jurisdictions.	8	0.49
8.1.3	PROMs data should be reported at the **individual level** (e.g. scores and changes in responses) only to the patient and/or to the patient's treating clinician/team. These data should be presented in a readily understandable format for the patient.	8	0.75
8.1.4	**PROMs data can be shared** through publications distributed directly to stakeholders, in research journals, and at conferences and forums. Audiences include all interested stakeholders, including clinicians and patients, to inform outcomes at the health service level, and funders and service providers, to inform policy and practice.	7	0.67
8.1.5	When using PROMs data for **benchmarking** purposes, disclosure or anonymity of centres/sites should be agreed on prior to publication.	7.5	0.56
**8.2**	**Access and data sharing**		
8.2.1	**De-identified** case-level data should be available for research purposes.	8	0.27
8.2.2	To encourage participation in PROMs collection and provide opportunities to maximise use of data, **clinicians should have access** to their patients’ PROMs data.	7	0.61
8.2.3	PROMs data sharing should be in accordance with **privacy legislation** and the registry data access policy.	9	0.24
**8.3**	**Timing and Frequency**		
8.3.1	**Timing and frequency of the PROMs reports** should be determined in advance and be in line with the data analysis plan.	7	0.72

*PROMIS* Patient-Reported Outcomes Measurement Information System, *PROQOLID* Patient-Reported Outcomes and Quality of Life Instruments Database

### Recommendations

The recommendations embedded within the domains of the recently published PROMs conceptual framework [16] are summarised below.

#### Rationale

This domain comprises two categories: “Purpose of collecting PROMs” and “Stakeholders*”*, each containing three recommendations. In this domain, registry users are introduced to the purpose and role of collecting PROMs within CQRs, including the variety of purposes and reasons of how, when, and why PROMs should be captured within registries. For example, *Use of PROMs should be driven by outcomes capable of contributing to improving patient care that can be met through patient reporting* (Recommendation 1.1.3). This domain also noted the wide range of potential stakeholders who may be interested in PROMs outcomes, including clinicians, health services, patients and researchers from different jurisdictions. The primary goals identified for PROM implementation may be dependent on the stakeholders engaged at the time of development.

#### Setting

The second domain focusses on PROMs implementation (two recommendations) and population and sample size (four recommendations). For example, the panel members highly agreed that, *Consideration should be given to piloting PROMs implementation before the full rollout to assess feasibility from the patient, clinician and/or system perspective* (2.1.2).

Recommendations identified that it was not always necessary to capture PROMs from all patients in the registry: *Depending on the purpose of data collection, PROMs may be collected from the whole population or a particular sample population* (2.2.4). In addition, the eligible population should be identified prior to the intervention: *consideration should be given to a screening process to identify the eligible population prior to the intervention* (2.2.2).

#### Ethics

There were only two recommendations under this domain. They were highly rated by panel members and focused on participant consent: *Information about the PROMs should be provided to participants using a method approved by an ethics committee, where the benefits and risks of participation are made clear, and includes how they can withdraw from participation at any time* (3.1.1) and *depending on the jurisdiction and institutional ethics review, PROMs may require an opt-in or opt-out approach* (3.1.2).

#### Instruments

This domain comprised three categories: “Consumer engagement”, “New/Existing” and “Generic/Specific” and contained 15 recommendations. The “Consumer engagement” category recommended the inclusion of patients when setting PROMs objectives and instrument choice, including the development and validation of new instruments, as well as use of PROMs data (4.1.1), instrument type and mode of administration for patients with special needs (4.1.3). It also suggests that *PROMs data collection should consider patient preferences for providing PROMs responses, including multiple modes of administration* (4.1.4).

The “New/Existing” category focusses on selection of PROMs in the CQRs. Recommendation 4.2.1 suggests that *PROMs selection should meet the purpose of implementation and reflect the identified outcome of interest*, and that *PROMs selection should commence with a literature review to identify the range and frequency of use of existing validated instruments*. In addition, registries need to ensure that *New and abbreviated PROMs need to be evaluated for validity before being used to measure and report on outcomes* (4.2.3). It is essential for *PROMs to be translated into multiple languages depending on the availability of validated translated versions and the cost of implementation* (4.2.5). Finally, *PROMs selection should be based on recommendations by experts in the field including patients with lived experience of the disease/condition* (4.2.7).

A further recommendation was that registries should consider using item banks (repositories of validated QoL questions) (recommendation 4.3.1) (e.g. PROMIS [28], PROQOLID [29]). Sometimes multiple instruments could be included in the registry: *More than one instrument may be required to meet the objectives of the PROMs data collection* (4.3.3). Generic instruments may be useful in a registry setting for global health, research and policy purposes to compare against outcomes from other populations or healthcare interventions. Condition-specific PROMs have greater clinical utility related to particular conditions, treatments and procedures (4.3.2).

#### Administration

This domain comprises 10 recommendations, four under the “Timing and Frequency” category and six under “Modes and Methods”. Recommendation 5.1.1 suggests PROMs should be administered at various time points (e.g. baseline, single or multiple). These need to be based on discipline-specific clinical best practice and evidence. *The length of PROM data collection tools and numbers of data collection points should consider patient and administrative burden*” (5.1.2). In addition, “*processes should be developed to avoid sending follow-up PROMs to deceased patients or patients who have withdrawn their informed consent* (5.1.4).

Registries should outline plans for PROMs administration (e.g. paper, telephone, electronic, other) and setting (e.g. clinic, home, other) (5.2.1). Patient factors, such as *age, gender and digital literacy* also need to be considered (5.2.4). To minimise the burden of data collection, *Computer adaptive testing systems should be considered to minimise patient’s time and data entry burden* (5.2.5).

#### Data management

Three recommendations under the “Entry and Quality checks” category were rated highly by panel members. For example, recommendation 6.1.1 states that *PROMs data management should consider issues of data security, information governance, and availability of technology for data collection.* Two recommendations were developed on data management protocols, e.g. *Registries should provide data management protocols and training to assist staff in PROMs administration, data collection and data entry* (6.2.3).

In terms of information technology (IT) and data storage, *PROMs IT modules should be designed to support PROM completion, such as sending regular email or phone reminders, and validation checks for missing data prior to submission, and should store the completed data with the date of completion*” (6.2.1). In addition, an *appropriate strategy should be developed for managing missing PROMs data* (6.2.3).

#### Statistical methods

This domain consisted of eight recommendations focusing on statistical methods and analysis of PROMs data. The first five recommendations were highly rated by panel members. For example, recommendation 7.1.1 suggests for *biostatisticians and/or epidemiologists to be involved in processing and reporting PROMs data*. It is crucial that *methods for data analysis and the volume, nature, and management of missing PROMs data are clearly described* (7.1.3).

The remaining recommendations address analysis of baseline and follow-up data (7.1.5), risk adjustment to control for *the role of confounding and case mix in PROMs data analysis* (7.1.6), *adjusting confounding and case-mix factors* (7.1.7) *and real-time analysis for shared decision making* (7.1.8).

#### Feedback and reporting

This domain comprised three sections and nine recommendations on feedback and reporting of PROMs data. Five recommendations were included under the “Dissemination” category. This category focuses on stakeholders and suggests that PROMs *output should be published in a range of formats to reach a broad range of stakeholders* (8.1.1). Recommendation 8.1.4 states the following: *Audiences include all interested stakeholders, including clinicians and patients, to inform outcomes at the health service level, and funders and service providers, to inform policy and practice* (8.1.4). Individual PROMs data reporting should be available *only to the patient and/or to the patient’s treating clinician/team* (8.1.3).

“Access and data sharing” had three recommendations. Recommendation 8.2.1 states that *de-identified case-level data should be available for research purposes*. To encourage clinicians to collect PROMs, they *should have access to their patients’ PROM data* (8.2.2). In terms of the timing of reporting, recommendation 8.3.1 suggests that *timing and frequency of the PROMs reports should be determined in advance and be in line with the data analysis plan*.

## Discussion

This was a novel study to investigate the role of PROMs in CQRs and to develop preliminary recommendations for PROMs inclusion in clinical registries. From 117 potential statements following round 2 and addition of new statements, 62 were proposed for inclusion to the final set of recommendations across eight domains: Rationale, Setting, Ethics, Instruments, Administration, Data management, Statistical methods, and Feedback and reporting.

Successful PROMs implementation in CQRs includes many challenges and requires clinical, operational, and analytic resources and expertise. Recommendations for PROMs implementation are critically important for registries to assure meaningful PROMs data capture, use, interpretation, and reporting [16]. The newly developed recommendations complement the PROMs framework for CQRs [16] and provide a set of guiding principles for implementing PROMs in CQRs to provide maximum value and best outcomes. These recommendations guide the user in a stepwise manner from conception through to operational considerations and reporting. These include rationale for PROMs data collection, setting (e.g. population size), ethics and consent arrangements, instrument selection, mode, method and frequency of administration, data management, statistical methods for PROMs data collection, and feedback and reporting of the data.

The addition of PROMs into CQRs can be used to maximise the benefits of current registry objectives, or alternatively the addition of PROMs may extend the registry’s scope and enhance the registry’s utility [9]. Registries should consider piloting PROMs implementation before the full rollout to assess feasibility and sustainability from the patient, clinician and/or system perspective. A sustainable approach to using the PROMs may require significant long-term commitment of budget, resources to build a coherent system, and active support from diverse organisations [30].

With regards to instruments adopted, the panel recommended registries include both generic instruments (designed for use among diverse populations with a broad range of medical conditions) and condition-specific instrument/s, translated into multiple languages as needed. PROMs selection should be based on recommendations by experts in the field, as well as completion time for patients, license and administration costs and overall patient burden.

Previous studies demonstrated a number of issues relating to the administration and response rates of PROMs in registries [21, 31]. In our study this was reflected in the expert consensus that PROMs should be administered via multiple methods to increase response rate. In regards to the timing of data collection, it is recommended that PROMs be administered at various time points (e.g. baseline, single or multiple time points).

The experts in our study agreed that PROMs data should be shared with various stakeholders, including patients and clinicians to inform outcomes at the health service level; and funders and service providers, to inform policy, practice and reimbursement of healthcare services. Future research should examine how PROMs completion and feedback develops, and is in turn influenced by, the process of building relationships with patients, in addition to the impact of PROMs collection on information exchange and decision making. It is also important to consider ethical issues and purpose of the PROMs data access, management of concerning PROMs data, so that participants’ information is not be released outside without the permission of the participant [32].

This was the first study to develop preliminary recommendations for PROMs inclusion in CQRs using a Delphi consensus process [26]. Although the study recruited international participants, the Delphi panel was dominated by participants from Australia, which may limit the generalisability of the recommendations. Therefore, these recommendations may not be as relevant to other jurisdictions outside Australia. Despite there being no strict guidelines for sample size in a Delphi study, a relatively small panel size was another limitation of our study. A minimum sample size of 10 is usually recommended to obtain enough information and make valid conclusions of the research study [33]. Panels of similarly trained experts provide effective and reliable utilization of a small sample from a limited number of experts in a field of study to develop reliable criteria and recommendations that support effective decision-making [34].

The study would also be strengthened by including patients and other consumers with an interest in CQRs. Patient involvement with greater diversity will be important for future work and implementation of the recommendations.

Recommendations that are both practicable and robust in the interpretation of an evidence base can be achieved with the Grading of Recommendations Assessment, Development and Evaluation (GRADE) approach (also known as a method of assessing the certainty in evidence and the strength of recommendations in healthcare) [35, 36]. Due to limited literature in this area, it would not be appropriate to use GRADE on our recommendations.

## Conclusions

The recommendations for PROMs implementation in CQRs represent a valuable resource that can be used for educating registry managers, researchers and clinicians on the effectiveness of collecting, analysing and acting upon PROMs data to improve health outcomes, and to support PROMs implementation and use.

Developing preliminary recommendations is an important first step, as is supporting PROMs collection and reporting in CQRs and finally, for evaluating key patient outcomes. Next steps will involve testing and evaluation of the newly developed recommendations in the registry settings, which may lead to revisions of the recommendations from this study. Qualitative studies with registry managers and stakeholders including patients are planned to determine the utility and impact of the recommendations.” Further study involving international data custodians of large CQRs, QoL experts and PROMs specialists will be conducted to develop a user guide for PROMs inclusion in CQRs and a checklist for PROMs data collection and reporting in the registry setting.

## Data Availability

Data sharing not applicable as no datasets generated and/or analysed for this study.

## Abbreviations

CQRs: Clinical Quality Registries
DI: Disagreement Index
GRADE: Grading of Recommendations Assessment, Development and Evaluation
MI: Median Importance
QoL: Quality of Life
PROMs: Patient-Reported Outcome Measures
